# Familial Mediterranean Fever and COVID-19: Friends or Foes?

**DOI:** 10.3389/fimmu.2020.574593

**Published:** 2020-09-18

**Authors:** Alessandro Stella, Mohamed Lamkanfi, Piero Portincasa

**Affiliations:** ^1^Department of Human Oncology and Biomedical Sciences, University of Bari Aldo Moro, Bari, Italy; ^2^Department of Internal Medicine and Pediatrics, Ghent University, Ghent, Belgium; ^3^Division of Internal Medicine, Clinica Medica “A Murri”, Department of Biomedical Sciences and Human Oncology, University of Bari Aldo Moro, Bari, Italy

**Keywords:** FMF disease, COVID-19, cytokine storm, pyrin, innate immunity

## Abstract

Familial Mediterranean Fever (FMF) and COVID-19 show a remarkable overlap of clinical symptoms and similar laboratory findings. Both are characterized by fever, abdominal/chest pain, elevation of C-reactive protein, and leukocytosis. In addition, colchicine and IL-1 inhibitors treatments that are effective in controlling inflammation in FMF patients have recently been proposed for off-label use in COVID-19 patients. Thus, FMF may resemble a milder recapitulation of the cytokine storm that is a hallmark of COVID-19 patients progressing to severe disease. We analyzed the sequence of the MEFV-encoded Pyrin protein – whose mutations cause FMF- in mammals, bats and pangolin. Intriguingly, although Pyrin is extremely conserved in species that are considered either a reservoir or intermediate hosts for SARS-CoV-2, some of the most common FMF-causing variants in humans are present as wildtype residues in these species. We propose that in humans, Pyrin may have evolved to fight highly pathogenic infections.

## Introduction

The World Health Organization reported a novel coronavirus on December 30, 2019 as the cause of a cluster of pneumonia cases in the city of Wuhan in the Hubei Province of China. Since then, the severe acute respiratory syndrome coronavirus 2 (SARS-CoV-2) has infected nearly 30 million individuals worldwide causing more than 900,000 deaths during the past 6 months of the COVID-19 pandemic. Among European countries, Italy has been severely hit by COVID-19 (1). Since the initial outbreak, a huge body of clinical and scientific information has been accumulated on COVID-19, a multifaceted disease hitting not only lungs, but also other organs, with different defined stages (2, 3). In most cases, SARS-CoV-2 enters the human body through inhaled droplets and aerosols. Although contact with contaminated surfaces has been hypothesized as a second possible infection route, the importance of this alternative mode of infection has not been assessed systematically (4, 5). Upon infection, SARS-CoV-2 enters its target cells via: (a) binding of its spike protein (S) to angiotensin converting enzyme 2 (ACE2); (b) activation through proteolysis of the viral S protein catalyzed by the cellular transmembrane protease serine 2 (TMPRSS2); and (c) fusion of SARS-CoV-2 virus with the host cell membrane. A wide variation in allele frequencies at ACE2 expressions single nucleotide polymorphisms (eSNPs) loci can partly explain the differences in COVID-19 prevalence across different countries (6). Also, differential expression of ACE2 occurs in several human cancers and chronic diseases, possibly influencing COVID-19 susceptibility and severity (7). The involvement of ACE2 and TMPRSS2 in viral cell entry has been exploited to plan experimental therapies based on using protease inhibitors such as camostat mesylate or nafamostat to block SARS-CoV-2 entry into cells (8, 9). Once inside the host cells, the SARS-CoV-2 positive single stranded RNA (ssRNA) genome begins replication and cytoplasmic accumulation. The ssRNA or its double stranded intermediate (dsRNA) are recognized by the innate immune nucleic acid sensing systems whose activation exerts a first antiviral response through the production of type 1 interferons and the secretion of pro-inflammatory cytokines. The immunomodulation at this early stage of infection might determine the growth of the viral load, with most infected individuals still being asymptomatic or paucisymptomatic, while almost 15% of SARS-CoV-2 positive patients develop fever, coughing, occasionally ageusia and anosmia and even gastrointestinal symptoms (3), with or without hepatic involvement (10).

Patients progressing to the second phase show a strong immunological and hyperinflammatory response that is defined as a “cytokine storm” and which may lead to respiratory worsening and bilateral pneumonitis. In this second stage, COVID-19 patients manifest symptoms mimicking those present in patients with auto-inflammatory diseases such as fever, arthralgia, leucopenia and myocarditis (11–13).

## The Parallel Worlds of FMF and COVID-19

We were intrigued by the remarkable overlap between these clinical manifestations and some of the typical manifestations of Familial Mediterranean Fever (FMF), a largely recessively inherited monogenic inflammasomopathy (autoinflammatory disorder involving the inflammasome) caused by mutations in the *MEFV* gene that is particularly prevalent in the Mediterranean basin (14). While the previously mentioned clinical signs are not specific to FMF and shared with other hereditary recurrent fevers and inflammatory diseases, yet they represent an indication of similarities among FMF pathogenesis and the hyperinflammatory response observed in COVID-19 patients. Worthy of note, taste alteration has been reported amongst the prodromal manifestations preceding fever attacks in FMF (15, 16). We should stress that the cytokine storm observed in the inflammatory stage of COVID19, has been reported in other autoinflammatory diseases (AIDs) such as the macrophage activation syndrome (MAS) and adult onset Still’s disease (17). However, authors have often reported genetic heterogeneity and overlap between these AIDs and hereditary recurrent fevers (18–20).

Colchicine, a natural alkaloid from *Colchicum autumnale*, has a long history as a drug to treat pain and swelling since ancient Egypt. It is nowadays used to treat FMF, gout, Behçet syndrome and recurrent non-infective pericarditis. Unsurprisingly, it is currently being investigated in several COVID-19 therapeutic trials (21–23). Notably, in colchicine-resistant or colchicine-intolerant FMF patients, alternative treatments include biologics that neutralize the pro-inflammatory cytokine interleukin (IL)-1β directly (Canakinumab) or inhibit IL-1β-mediated activation of the IL-1 Receptor (Anakinra and Rilonacept) (24–26). Similarly to colchicine, several ongoing trials are evaluating the use of IL-1 pathway inhibitors to treat COVID-19 patients (27–30). Although first results from COVID-19 patients treated with these repurposed drugs have been conflicting (31–33), the role of Pyrin (the inflammasome sensor protein that is encoded by *MEFV*) in modulating severity and outcome of COVID-19 is still unknown.

## The Pyrin Inflammasome

The Pyrin domain architecture shows intriguing features which may add insights into its possible role in COVID-19 disease. Pyrin has a N-terminal pyrin domain (PYD) that is frequently found in other innate immune pathogen sensors that mount inflammasome responses such as NLRP1, NLRP3, and AIM2. The N-terminal PYD domain engages in homotypic interactions with its PYD counterpart in the adaptor protein apoptosis-associated speck-like protein with a caspase recruitment domain (ASC) to assemble ASC specks, which recruit the inflammatory protease procaspase-1 inducing its self-cleavage and activation (34). Caspase-1 in turn matures the proinflammatory cytokines IL-1β and IL-18 and cleaves gasdermin D to trigger a lytic cell death mode termed pyroptosis that promotes secretion of aforementioned cytokines along with danger-associated molecular patterns (DAMPs) such as IL-1alpha, HMGB1 and ATP (35).

Pyroptosis is a double-edged sword with both antiviral and proviral activities during viral infections (36). In fact, cell death can lead to halting viral replication and infection, frequently at the price of increased inflammation. Conversely, dead cells release a large number of viral particles contributing to viral dissemination.

The *MEFV* encoded Pyrin sensor contains in its central region three domains: a bZIP domain (aa 370-412), a B-box domain (aa 370-412) and a coiled-coil domain (CC, aa 420-440). The role of these three domains has not been thoroughly investigated and few FMF-causing variants localize to Pyrin’s central region (37). This region may have an autoinhibitory role precluding the PYD domain from interacting with ASC and activating pyroptosis (38). The C-terminal B30.2 (also known as PRY/SPRY) domain is extremely important in FMF pathogenesis since most of the disease-penetrant *MEFV* variants cluster in this region (39). Although this uneven distribution of FMF-associated *MEFV* mutations suggests that the B30.2 domain is crucial in regulating Pyrin inflammasome activity, the precise molecular mechanisms by which the B30.2 domain regulates FMF pathogenesis have not been elucidated (40). Likely, FMF-causing mutations in the B30.2 domain may derail intramolecular interactions that keep Pyrin in an autoinhibitory state.

## The Curious Family of Pyrin Orthologs

**FIGURE 1:**
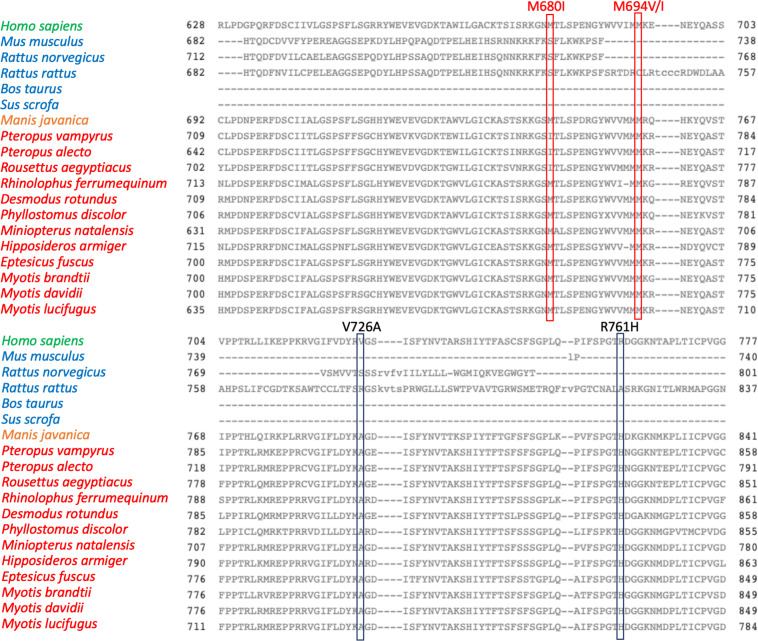
Alignment of 19 *MEFV* orthologs from top to bottom as follow: *Homo sapiens* NP_000234.1, *Mus musculus* NP_001155263.1, *Rattus norvegicus* XP_017452974.1, *Rattus rattus* XP_032769237.1, *Bos taurus* XP_015315767.1, *Sus scrofa* XP_013851182.1, *Manis javanica* XP_017515721.1, *Pteropus vampyrus* XP_011374846.1, *Pteropus alecto* XP_006913958.1, *Rousettus aegyptiacus* XP_015977766.1, *Rhinolophus ferrumequinum* XP_032957625.1, *Desmodus rotundus* XP_024409044.1, *Phyllostomus discolor* XP_028366066.1, *Miniopterus natalensis* XP_016066853.1, *Hipposideros armiger* XP_019488407.1, *Eptesicus fuscus* XP_028001472.1, *Myotis brandtii* XP_014400628.1, *Myotis davidii* XP_015414020.1, *Myotis lucifugus* XP_023611041.1. The common names for species analyzed are as follow: *M. musculus* (mouse), *R. norvegicus* (Norway or brown rat), *R. rattus* (black rat), *B. taurus* (cattle), *S. scrofa* (pig), *M. javanica* (Malayan pangolin), *P. vampyrus* (Large flying fox), *P. alecto* (black flying fox), *R. aegyptiacus* (Egyptian rousettes or Egyptian fruit bat), *R. ferrumequinum* (greater horseshoe bat), *D. rotundus* (common vampire bat), *P. discolor* (pale spear-nosed bat), *M. natalensis* (Natal long-fingered bat), *H. armiger* (great roundleaf bat), *E. fuscus* (big brown bat), *M. brandtii* (Brandt’s bat), *M. davidii* (David’s myotis), *M. lucifugus* (little brown bat). Alignment were performed using the Cobalt software (Papadopoulos JS and Agarwala R, Bioinformatics 23:1073-79, 2007). Boxes highlight the amino acid residues corresponding to common FMF-causing variants. In green homo sapiens Pyrin, in blue pyrin from mammal species, in orange pangolin Pyrin, in red bats pyrin proteins.

Interestingly, the degree of amino acid conservation along the pyrin protein sequence is rather variable and may offer some insights in the dangerous liaisons between FMF and COVID-19. We aligned the *MEFV-*encoded Pyrin amino acid sequences from 19 different species including the only pangolin and all bat species sequences available in GenBank (Figure 1). Bats and pangolins have been considered the reservoir and intermediate host, respectively, of SARS-CoV-2 before transmission to humans.

The alignment of the Pyrin sequences presented unique evolutionary features (Figure 1).

In fact, some of the most prevalent FMF-associated mutations in human Pyrin were present as wild type in all bat species analyzed and in pangolin (V726A, R761H). This resembles previous findings that some FMF-associated mutations are retrieved as wild type in primates, suggesting evolutionary pressure on Pyrin (41). In contrast, other FMF-associated amino acids residues that are largely prevalent in middle eastern populations (M680I, M694I, M694V) were not observed in bats and pangolin.

Worthy of note M680I, M694V, M694I, and R761H were all associated with derailed Pyrin-induced IL-1β secretion in a recently developed blood-based functional test for FMF alleles (42). It is tempting to speculate that FMF patients carrying V726A and R761H variants- which represents the wild type residues in all bats and pangolin sequences- might modulate better their cytokine response to SARS-CoV-2 infection. Further, in a mouse knock-in model the V726A variant causes a more severe FMF phenotype compared to M694V, M680I, and elevated production of multiple cytokines (43). Given that the M680I and M694V/I alleles in patients provoke a hyper-inflammatory response not different from V726A and R761H (at least in FMF), one may hypothesize that they could warrant a comparable attenuation of viral infection. An elaboration on this hypothesis would stem from the increased FMF severity associated to M680I and M694V/I mutations. This raised level of inflammation can either move the balance toward excess inflammation in COVID-19 or be causing an even improved immunomodulation in COVID-19 compared to V726A, R761H. Moreover, historic pandemics and different pathogens may have selected for different *MEFV* variants in their respective host species and populations. Thus, a pathogen different from coronaviruses (i.e., *Yersinia pestis*) might have selected the M680I, M694V/I mutations in humans but not in bats. Indeed, confirming this hypothesis, and shortly after the submission of this work, genetic and experimental evidences have been reported linking these *MEFV* variants with resistance to *Yersinia pestis* (44, 45).

**FIGURE 2:**
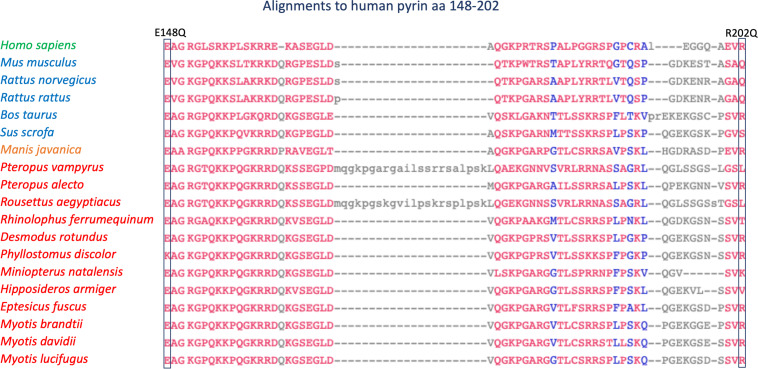
Alignment of 19 Pyrin orthologs from amino acid 148 to amino acid 202 (human gene). Alignment order and color coding as in Figure 1.

The E148Q and R202Q *MEFV* variants, which according to a current consensus are considered neutral polymorphisms, showed a response to colchicine challenge alike normal controls (42). These two variants presented rather divergent evolutionary features. While the glutamic acid at position 148 was strictly conserved in all species analyzed, the arginine at position 202 was conserved in pangolin and in 7 of 13 bat species, and changed to a glutamine in mouse and rats (Figure 2). Therefore, E148Q and R202Q which appear to be neutral in functional assays seem to be under apparently different evolutionary pressures.

## The Less than Casual Worldwide Distribution of Pyrin Variants

The frequency of *MEFV* mutations and polymorphisms shows a great variability in countries where FMF has a high frequency. In fact, the M694V and M694I, are prevalent among Turks, non-ashkhenazi Jews, and Arabs, while the M680I is frequent in the Armenian population (46–49). The V726A and R761H are generally associated with a milder phenotype, and generally reported in clusters of FMF patients of Ashkhenazi Jewish origin, and in the western Mediterranean area (50). The E148Q variant, whose pathogenicity is still debated, also presents a wide variation in frequency across different populations (51).

This peculiar distribution of the *MEFV* variome led to the hypothesis that the *MEFV* gene has been subjected to a balancing selection and nucleotide variation, particularly in the B30.2 region, is adaptive (52). Thus, the severity of COVID-19 disease in FMF patients, once infected, might be influenced, at least partially, depending on specific *MEFV* genotypes which shows country-specific differences.

**FIGURE 3:**
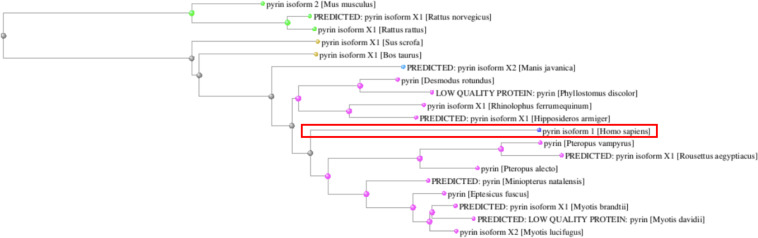
Phylogenetic tree of the Pyrin orthologs reconstructed from the COBALT multiple sequence alignment tool. The human Pyrin is boxed.

The *MEFV* gene displays other intriguing evolutionary features. In fact, while the N-terminal PYD domain is conserved across all species, the B30.2 domain appears of more recent origin. Of note, when considering the entire Pyrin amino acid sequence, human Pyrin is more closely related to its bats and pangolin homologs than to other mammal species (Figure 3).

**FIGURE 4:**
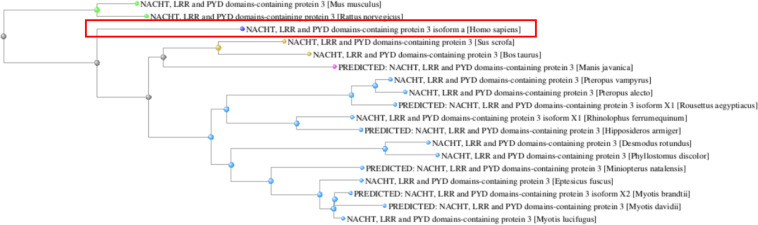
Phylogenetic tree of the NLRP3 orthologs reconstructed from the COBALT multiple sequence alignment tool. The human NLRP3 protein is boxed.

In contrast, the human NLRP3 protein presented a high level of homology in all analyzed species. It clustered with other mammalian NLRP3 proteins in the phylogenetic tree and was more distantly related to bat sequences (Figure 4).

**FIGURE 5:**
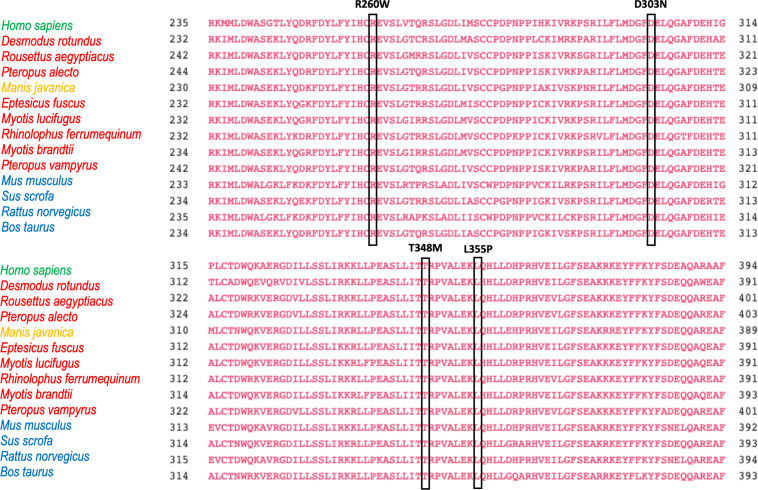
Alignment of 14 NLRP3 orthologs from amino acid 235 to amino acid 394 (human gene). The four residues whose mutations are responsible of more than 40% of NLRP3 –associated autoinflammatory diseases are boxed.

In addition, differently from *MEFV*, the most common human *NLRP3* mutations- R260W, D303N, T348M, and L355P- which represent more than 40% of the total mutation burden for this gene (53, 54), were never present as wild type amino acid residues in all bat species and pangolin (Figure 5).

Therefore, considering the Pyrin inflammasome a passive bystander in SARS-CoV-2 infection could lead to overlooking an important innate immune pathway that coordinates the response to pathogens. In fact, inflammasome-driven pyroptosis is one of the results of the cytokine storm which concurs to the high pathogenicity of both SARS-CoV, and SARS-CoV-2 (55, 56).

## The Role of the NLRP3 Inflammasome in Viral Infections

Recent findings demonstrated that the SARS-CoV ORF3a protein can provoke a cytokine storm by activation of the NLRP3 inflammasome through TRAF3-dependent ubiquitination of ASC (57). A recent analysis of 2782 SARS-CoV-2 strains showed that non-synonymous substitutions in the SARS-CoV-2 ORF3a protein may alter virulence and infectivity (58). Additional viral proteins are able to activate the NLRP3 inflammasome. The NLRP3-mediated response to influenza A virus (IAV) has been extensively studied and plays a crucial role in protecting the host while helping in clearing the infection. However, if the inflammatotory response is particularly prolonged and excessive it can increase disease burden (59). The NLRP3 inflammasome plays a critical role in guarding against IAV infection and reducing lung damage consequent to infection (59, 60). Other viruses are capable of activating the NLRP3 inflammasome. The viroporin 2B, released upon human rhinovirus infection (HRV), causes proteolytic activation of procaspase-1 and IL-1ß secretion in a NLRP3 dependent manner (61). Several other viruses can provoke a sustained response from the NLRP3 inflammasome as extensively reviewed (62). Similarly to the *MEFV*-encoded Pyrin, the N-terminal PYD domain of NLRP3 can recruit ASC via homotypic interaction with its PYD counterpart on ASC to assemble ASC filaments. The assembly of ASC filaments in macromolecular structures known as ASC specks allow the recruitment of procaspase-1 to induce its self-cleavage and activation. Caspase-1 in turn cleaves the proinflammatory cytokines IL-1β/IL-18 and gasdermin D to trigger pyroptosis. Thus, both Pyrin and NLRP3 inflammasomes may compete for ASC binding and ASC oligomerization-dependent caspase-1 activation.

## And the Answer Is….

**FIGURE 6:**
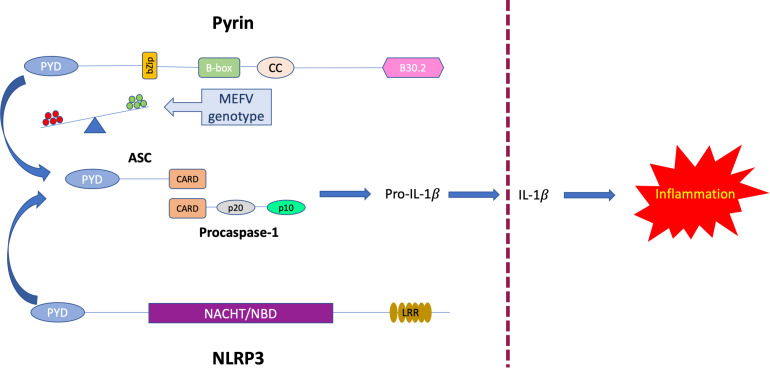
Proposed model of competitive binding to ASC of *MEFV*-encoded Pyrin and NLRP3. Abbreviations used: PYD, Pyrin domain, CARD, Caspase recruitment domain, NACHT/NBD NACHT-, Nucleotide binding- domain, LRR, Leucine-rich repeats.

Before the COVID-19 pandemic, a recent report (63) demonstrated that bats, when infected with different zoonotic viruses, can sustain high viral loads while presenting a dampened NLRP3-mediated inflammatory response associated with a decrease in both ASC speck formation and IL-1β secretion. Hence, a dysregulation of the NLRP3 inflammasome can also contribute to the utterly complex individual immune response to viral infections (64). These findings suggest that the modulation of innate immunity rather than an enhanced antiviral defense might shape the different outcome of COVID-19 disease. Thus, we hypothesize that competition between Pyrin and the NLRP3 inflammasomes for ASC recruitment may tilt the balance between a cytokine storm and a finely adjusted protective inflammation (Figure 6).

FMF, in which Pyrin activity and consequent ASC oligomerization are increased because of *MEFV* pathogenic variants, may therefore represent a unique opportunity as a disease model to investigate the regulation of the inflammatory response to novel emerging viruses. We presented here the unique evolutionary features of the *MEFV*-encoded Pyrin suggesting its putative contribution in shaping the individual risk to develop severe complications consequent to infectious diseases.

Several factors could have contributed to the rapid spreading of COVID-19 pandemic, including access to adequate health care, aging demographic, metabolic dysfunctions, socio-cultural differences. It would be wise not to discard individual genetics, including the *MEFV* gene, from the mix.

Future investigation on how carriers of different *MEFV* genotypes have responded to coronavirus infections could help in refining existing and novel therapeutics in development for present and future challenges. In conclusion, the answer to the title question might not be blowing in the wind but could hopefully be found in a deeper knowledge of inflammasome regulation in well-known inflammatory diseases.

## Data Availability Statement

The datasets presented in this study can be found in online repositories. The names of the repository/repositories and accession number(s) can be found in the article/ Supplementary Material.

## Author Contributions

AS designed the study and drafted the manuscript. ML and PP drafted the manuscript. All authors contributed to the article and approved the submitted version.

## Conflict of Interest

The authors declare that the research was conducted in the absence of any commercial or financial relationships that could be construed as a potential conflict of interest.
