# Supplementary material for: Familial Mediterranean Fever and COVID-19: Friends or Foes?
Source: Front Immunol. 2020 Sep 18;11:574593. doi: 10.3389/fimmu.2020.574593 (PMC7530822; doi:10.3389/fimmu.2020.574593)
Supplement: Supplementary file 1 [file Table_1.DOCX]

**SUPPLEMENTARY FILE 1**

Sequences used for alignment and building of the Pyrin phylogenetic tree.

*Homo sapiens* NP_000234.1, *Mus musculus* NP_001155263.1*, Rattus norvegicus* XP_017452974.1*, Rattus rattus* XP_032769237.1*, Bos taurus* XP_015315767.1*,* *Sus scrofa* XP_013851182.1, *Manis javanica* XP_017515721.1, *Pteropus vampyrus* XP_011374846.1, *Pteropus alecto* XP_006913958.1, *Rousettus aegyptiacus* XP_015977766.1, *Rhinolophus ferrumequinum* XP_032957625.1, *Desmodus rotundus* XP_024409044.1*, Phyllostomus discolor* XP_028366066.1*, Miniopterus natalensis* XP_016066853.1, *Hipposideros armiger* XP_019488407.1, *Eptesicus fuscus* XP_028001472.1, *Myotis brandtii* XP_014400628.1, *Myotis davidii* XP_015414020.1, *Myotis lucifugus* XP_023611041.1.

Sequences used for alignment and building of the NLRP3 phylogenetic tree.

*Homo sapiens* NP_001230062.1, *Mus musculus* NP_665826.1*, Rattus norvegicus* NP_001178571.1*, Bos taurus* NP_001095689.1*,* *Sus scrofa* NP_001243699.1, *Manis javanica* XP_017529157.1, *Pteropus vampyrus* XP_011383771.1, *Pteropus alecto* XP_024896314.1, *Rousettus aegyptiacus* XP_015980967.1, *Rhinolophus ferrumequinum* XP_032951655.1, *Desmodus rotundus* XP_024416695.1*, Phyllostomus discolor* XP_028390193.1*, Miniopterus natalensis* XP_016058924.1, *Hipposideros armiger* XP_019489267.1, *Eptesicus fuscus* XP_008149763.1, *Myotis brandtii* XP_005853233.1, *Myotis davidii* XP_006764378.1, *Myotis lucifugus* XP_023619604.1.

Alignments were performed using the Cobalt software (Papadopoulos JS and Agarwala R, Bioinformatics 23:1073-79, 2007 PMID: 17332019).
